# Are bacteria, fungi, and archaea present in the midtrimester amniotic fluid?

**DOI:** 10.1515/jpm-2022-0604

**Published:** 2023-05-17

**Authors:** Roberto Romero, Maria Teresa Gervasi, Daniel B. DiGiulio, Eunjung Jung, Manaphat Suksai, Jezid Miranda, Kevin R. Theis, Francesca Gotsch, David A. Relman

**Affiliations:** Pregnancy Research Branch, Division of Obstetrics and Maternal-Fetal Medicine, Division of Intramural Research, Eunice Kennedy Shriver National Institute of Child Health and Human Development, National Institutes of Health, United States Department of Health and Human Services (NICHD/NIH/DHHS), Bethesda, MD, and Detroit, MI, USA; Department of Obstetrics and Gynecology, University of Michigan, Ann Arbor, MI, USA; Department of Epidemiology and Biostatistics, Michigan State University, East Lansing, MI, USA; Center for Molecular Medicine and Genetics, Wayne State University, Detroit, MI, USA; Detroit Medical Center, Detroit, MI, USA; Gynaecology and Obstetrics Unit, Department of Woman and Child Health, University Hospital of Padua, Padua, Italy; Department of Medicine, Stanford University School of Medicine, Stanford, CA, USA; Infectious Diseases Section, Veterans Affairs Palo Alto Health Care System, Palo Alto, CA, USA; Department of Obstetrics and Gynecology, Wayne State University School of Medicine, Detroit, MI, USA; Department of Obstetrics and Gynecology, Universidad de Cartagena, Cartagena, Colombia; Department of Biochemistry, Microbiology, and Immunology, Wayne State University School of Medicine, Detroit, MI, USA; Department of Microbiology and Immunology, Stanford University School of Medicine, Stanford, CA, USA

**Keywords:** chorioamnionitis, contamination, inflammation, microbial survey, sterile womb hypothesis

## Abstract

**Objectives:**

This study was conducted to determine whether bacteria, fungi, or archaea are detected in the amniotic fluid of patients who underwent midtrimester amniocentesis for clinical indications.

**Methods:**

Amniotic fluid samples from 692 pregnancies were tested by using a combination of culture and end-point polymerase chain reaction (PCR) techniques. Intra-amniotic inflammation was defined as an interleukin-6 concentration >2,935 pg/mL.

**Results:**

Microorganisms were detected in 0.3% (2/692) of cases based on cultivation, 1.73% (12/692) based on broad-range end-point PCR, and 2% (14/692) based on the combination of both methods. However, most (13/14) of these cases did not have evidence of intra-amniotic inflammation and delivered at term. Therefore, a positive culture or end-point PCR in most patients appears to have no apparent clinical significance.

**Conclusions:**

Amniotic fluid in the midtrimester of pregnancy generally does not contain bacteria, fungi, or archaea. Interpretation of amniotic fluid culture and molecular microbiologic results is aided by the assessment of the inflammatory state of the amniotic cavity. The presence of microorganisms, as determined by culture or a microbial signal in the absence of intra-amniotic inflammation, appears to be a benign condition.

## Introduction

The amniotic cavity is thought to be sterile, a concept consistent with the “sterile womb hypothesis,” which posits that under normal circumstances microbial colonization occurs after birth [1], [2], [3]. Some investigators have reported the presence of microorganisms when using DNA sequencing techniques and proposed that human amniotic fluid contains evidence of a microbiome [4], [5], [6], [7], [8], [9], [10], [11], even in clinical states of health. These findings contrast with those of others who used either cultivation or molecular microbiologic techniques, or a combination of both, and did not find evidence of a microbiome [12], [13], [14], [15], [16], [17], [18]. The present study was conducted to determine whether bacteria, fungi, or archaea could be found in the amniotic fluid of patients who underwent midtrimester amniocentesis for clinical indications (see Table 1).

**Table 1:** Clinical characteristics, socio-demographics, and perinatal outcomes of the study population.

Characteristics	n=692
	n, or median (IQR)
Maternal age, years	37 (35–39)
Maternal race	
Caucasian	683 (98.7)
Black	3 (0.43)
Hispanic	3 (0.43)
Asian	3 (0.43)
Nulliparity	278 (40.2)
History of spontaneous preterm delivery	22 (3.2)
Gestational age at amniocentesis, weeks	16.3 (15.9–16.7)
Indication for amniocentesis	
Advanced maternal age	560 (80.9)
Abnormal 1st or 2nd trimester screening	63 (9.1)
Maternal request	49 (7.1)
Suspected fetal anomaly at ultrasound	11 (1.6)
Others	9 (1.3)
Gestational age at delivery	39.6 (38.6–40.6)
Preterm birth (<37 weeks)	56 (8.1)
Late spontaneous preterm delivery (≥32 weeks)	37 (5.3)
Early spontaneous preterm delivery (<32 weeks)	4 (0.6)
Indicated preterm delivery	15 (2.16)
Delivery route	
Vaginal	524 (75.7)
Cesarean	168 (24.3)
Gender	
Male	338 (49.2)
Female	349 (50.8)
Birthweight, grams	3335 (3041–3600)
Birthweight percentile	43 (27.6–63.9)
Small for gestational age	42 (6.1)

IQR, Interquartile range. Missing data: Gender (5).

## Materials and methods

This retrospective cohort study included 692 asymptomatic women with a singleton pregnancy who underwent midtrimester amniocentesis for clinical indications between 14 and 26 weeks of gestation (Table 1). Amniotic fluid was cultured for both aerobic and anaerobic bacteria as well as for genital mycoplasmas. Broad-range end-point polymerase chain reaction (PCR) assays were performed to amplify and characterize the ribosomal DNA (rDNA) of bacteria, fungi, and archaea, using a method previously described in detail [19]. In seven cases, quantitative PCR [19] was performed to assess microbial burden. Amniotic fluid concentrations of interleukin (IL)-6 were measured and validated with specific enzyme-linked immunosorbent assays. Intra-amniotic inflammation was diagnosed when the amniotic fluid IL-6 concentration was >2,935 pg/mL. White blood cell count, glucose concentration, and Gram stain of amniotic fluid were also performed. Microbial invasion of the amniotic cavity was defined by the presence of a positive culture or of microbial sequences by broad-range PCR.

The collection of samples and clinical data was approved by the Institutional Review Board of the Azienda Ospedaliera Treviso, Azienda Ospedale/Universita’ Padova, Veneto Region, Italy. This institution has a Federal Wide Assurance with the United States Department of Health and Human Services. This study is based on a subset of samples previously investigated in intra-amniotic inflammation [12].

Categorical data are presented as n (%) and continuous data as mean [standard deviation (±SD)] or median [interquartile range (IQR)] according to their distribution. The Kruskal-Wallis test, followed by the Mann-Whitney-Wilcoxon test for post-hoc analysis, was performed to compare continuous variables among and between groups. Comparisons of proportions were performed with Chi-square or Fisher’s exact tests. Statistical analysis was performed by using R language and environment for statistical computing (www.r-project.org). For all analyses, a two-tailed p-value<0.05 was considered significant.

## Results

Bacteria were detected in 0.3% (2/692) of cases based on cultivation, 1.73% (12/692) based on broad-range end-point PCR, and 2% (14/692) based on the combination of culture and end-point PCR results (Table 2). Collectively, eight bacterial taxa, one fungal species (*Candida albicans*), and no archaea were identified. Two bacterial taxa (*Staphylococcus aureus* and *Pseudomonas aeruginosa*) were detected by culture only and six bacterial taxa (*Ureaplasma*, *Acinetobacter*, *Mycoplasma hominis*, *Streptococcus agalactiae*, *Streptococcus*, and *Sneathia*) were detected by PCR only. There was no concordance between the results of microbial culture and molecular microbiology. Importantly, quantitative PCR did not yield a signal above the limit of detection of the assay in five of the seven cases tested (Table 2).

**Table 2:** Amniotic fluid markers of inflammation and pregnancy outcomes in patients with a positive amniotic fluid culture or an end-point PCR for microorganisms during the midtrimester.

Case number	Microorganism identified by culture	Microorganism identified by end-point PCR	Microbial burden 16S rRNA gene copy number	AF WBC, cells/mm^3^	AF glucose, mg/dL	AF IL-6, ng/mL	GA at delivery, weeks
1	Negative	*Ureaplasma*	13,003	30	34	8.43	28.6
2	Negative	*Acinetobacter*	745	12	35	0.40	34.7
3	Negative	*S. agalactiae*	NI	1	51	0.58	41.3
4	Negative	*M. hominis*	NI	2	44	0.43	41
5	Negative	*Streptococcus*	Below limit of detection	5	45	0.60	41.4
6	Negative	*Sneathia*	NI	5	50	0.17	40.9
7	Negative	*Sneathia*	NI	2	44	0.48	39.1
8	Negative	*Candida*	Below limit of detection	7	54	0.20	41.1
9	Negative	*Candida*	NI	2	45	0.10	39.4
10	Negative	*Candida*	Below limit of detection	11	46	0.06	39.3
11	Negative	*Candida*	NI	1	55	0.41	38
12	Negative	*Candida*	NI	2	50	0.3	37
13	*Staphylococcus aureus*	Negative	Below limit of detection	2	50	0.87	40.1
14	*Pseudomonas aeruginosa*	Negative	Below limit of detection	3	37	1.09	39.0

AF, amniotic fluid; IL-6, interleukin-6; GA, gestational age; NI, no information; PCR, polymerase chain reaction; WBC, white blood cell count.

The prevalence of spontaneous preterm birth (<37 weeks of gestation) was 5.78% (40/692) whereas that of spontaneous early preterm birth (<32 weeks of gestation) was 1.15% (8/692). Sterile intra-amniotic inflammation (IL-6>2,935 pg/mL and an absence of detectable microorganisms) was found in 6.1% (42/692) of patients in the midtrimester of pregnancy. Patients whose amniotic fluid samples had microorganisms but not inflammation had a similar perinatal outcome to that of women without detectable microorganisms. None of the patients with a positive amniotic fluid culture had intra-amniotic inflammation, and all delivered at term. A positive PCR result was associated with intra-amniotic inflammation in only one case (Case #1 in Table 2), specifically a patient with a high amniotic fluid microbial burden of *Ureaplasma* (13,003 16S rRNA gene copies) detected only by PCR but not by cultivation. The patient delivered at 28.6 weeks of gestation.

## Discussion

Our findings indicate that most asymptomatic patients in the midtrimester of pregnancy do not have bacteria, fungi, or archaea detectable in amniotic fluid and these observations are consistent with previous reports [12], [13], [14], [15].

The standard diagnosis of amniotic fluid infection depends upon the identification of microorganisms in amniotic fluid obtained in a manner that minimizes the risk of contamination of the specimen (transabdominal amniocentesis vs. intrauterine pressure catheter) [20]. Cultures of aerobic and anaerobic bacteria have been the gold standard. Molecular microbiologic techniques, using PCR with primers designed to target the conserved regions of microbial genomes or primers for specific microorganisms, have emerged as complementary methods to cultures for microbial detection [21], [22], [23], [24], [25], [26], [27], [28]. The individual and combined use of these methods for the diagnosis of intra-amniotic infection has been the subject of previous publications [19, 21, 23], [24], [25], [26, 29], [30], [31], [32], [33], [34], [35], [36], [37], [38], [39]. In general, molecular microbiologic techniques are more sensitive than cultivation techniques [40], [41], [42], [43]. Cultivation assays are also prone to false positive results due to contamination of a specimen either at the bedside or in the laboratory [44], [45], [46].

In the current study, two cases of positive amniotic fluid cultures were identified. Neither patient showed clinical evidence of intra-amniotic inflammation (white blood cell count and concentrations of glucose and IL-6), and both had a negative PCR result for microorganisms and subsequently delivered at term. These findings suggest that the positive cultures in these cases represented contamination of the specimens. We have previously reported that a positive amniotic fluid culture in the absence of intra-amniotic inflammation is a benign condition associated with term delivery and normal outcome [47].

Herein, 12 patients had a positive end-point PCR result for microorganisms but only one showed evidence of intra-amniotic inflammation (Case #1 in Table 2). This patient had a preterm delivery at 28 weeks of gestation with histologic evidence of acute chorioamnionitis (a maternal inflammatory response) and funisitis (a fetal inflammatory response). These observations suggest that the patient had a true positive PCR test even though the amniotic fluid culture was negative. Of interest, the microbial burden was high with quantitative real-time PCR. Previous reports suggested that the microbial burden in amniotic fluid may be helpful in the differential diagnosis of a true positive vs. a false positive result of molecular microbiologic tests [25].

Quantification of the microbial burden with real-time PCR for bacterial 16S rDNA was performed in seven cases of which five were positive for the end-point PCR and two were positive by culture. In most cases, real-time PCR did not detect microbial nucleic acids. In one case, the microbial burden was high (13,003 16S rRNA gene copies), and this patient is described in the previous paragraph. In the second case, *Acinetobacter* was identified by end-point PCR but there were only 745 16S rRNA gene copies. This patient had no evidence of intra-amniotic inflammation (Case #2 in Table 2) and delivered at 34 weeks of gestation; the placenta showed no evidence of acute histologic chorioamnionitis or funisitis.

We conclude that amniotic fluid in the midtrimester of pregnancy of clinically asymptomatic patients generally does not contain bacteria, fungi, or archaea. Interpretation of amniotic fluid culture and molecular microbiologic results is aided by assessment of the inflammatory state of the amniotic cavity, and by the quantification of microbial sequences. The presence of microorganisms by culture or a microbial signature by end-point PCR in the absence of intra-amniotic inflammation appears to be a benign condition.
